# Cost-effectiveness of edaravone dexborneol versus edaravone for the treatment of acute ischemic stroke in China: Based on the TASTE study

**DOI:** 10.3389/fphar.2022.938239

**Published:** 2022-10-18

**Authors:** Fenghao Shi, Zixuan He, Lin Wang, Hang Su, Sheng Han

**Affiliations:** ^1^ International Research Center for Medicinal Administration, Peking University, Beijing, China; ^2^ School of Pharmaceutical Sciences, Peking University, Beijing, China; ^3^ School of International Pharmaceutical Business, China Pharmaceutical University, Nanjing, China

**Keywords:** cost effectiveness, acute ischemic stroke, edaravone, edaravone dexborneol, economic evaluation

## Abstract

**Background and purpose:** The TASTE trial indicated that patients with acute ischemic stroke (AIS) using edaravone dexborneol have a significantly higher proportion of 90-day good functional outcomes (mRS 0–1) than those using edaravone. This study compared the cost-effectiveness of the aforementioned interventions in treating AIS in the Chinese setting, aiming to inform treatment decisions in clinical practice.

**Methods:** A model combining a decision tree and a Markov model was developed to assess the cost-effectiveness of edaravone dexborneol *versus* edaravone for AIS over a 30-year time horizon from the Chinese healthcare system’s perspective. Both efficacy and safety data were extracted from the TASTE study. Local costs and utilities were derived from publications and open-access databases; both cost and effectiveness were discounted at a rate of 5% per year. Sensitivity analyses were conducted to ensure robustness and identify the main drivers of the result.

**Results:** Compared with edaravone, edaravone dexborneol for AIS was found to be cost-effective in the first year and highly cost-effective as the study time horizons extended. In the long term (30 years), edaravone dexborneol yielded a lifetime gain of 0.25 (0.07–0.45) quality-adjusted life years (QALYs) at an additional cost of CNY 2201.07 (-3,445.24–6,637.23), yielding an ICER of CNY 8823.41 per QALY gained under the willingness-to-pay (WTP) of 1.5 times per capita GDP (121,464 CNY). The result is robust in both deterministic and probabilistic sensitivity analysis (PSA) methods, with the advantage of the edaravone dexborneol strategy increasing over time. Specifically, the probability of edaravone dexborneol dominant dexborneol is 76.30%, 98.90%, and 99.50% over 1-, 5-, and 30-year time horizons.

**Conclusion:** Both short- and long-term economic analyses suggest that edaravone dexborneol is highly likely to be a cost-effective alternative to treat AIS compared with edaravone in China.

## Introduction

According to the Global Burden of Disease Study 2019, China bears the highest lifetime risk of stroke worldwide, with 28.76 million prevalent cases, 3.94 million new stroke cases, and 2.19 million deaths in 2019 (Wang et al., 2022). Stroke is also one of the leading causes of disability-adjusted life year (DALY) in China, estimated to be as high as 45.9 million in 2019 (Wang et al., 2022). Of all strokes, ischemic stroke accounts for 82% and results in over 30 billion CNY direct and indirect losses every year, placing a heavy burden on society, families, and individuals (Sun et al., 2021).

Existing research suggests that early reperfusion therapy, including intravenous thrombolysis (IVT) and endovascular therapy (EVT), is the most effective and cost-effective treatment for AIS (Lees et al., 2010; Pan et al., 2014; Berkhemer et al., 2015; Pan et al., 2018). However, early reperfusion therapy is not meeting clinical needs. According to a new study, the overall IVT rate for acute ischemic stroke (AIS) was 5.64%, and the EVT rate was 1.45% in China between 2019 and 2020 (Ye et al., 2022).

In addition to reperfusion therapy, neuroprotective agents, such as edaravone and (+)-borneol, are another effective treatment for stroke (Zhang et al., 2005; Watanabe et al., 2008; Liu et al., 2011; Wu et al., 2014). Edaravone dexborneol is a multi-target neuroprotective agent consisting of edaravone and dexborneol in a 4:1 ratio. A double-blind, randomized, controlled study [NCT02430350] among 1,200 patients with AIS showed that edaravone dexborneol has clear efficacy benefits (modified Rankin scale score (mRS) ≤1, 67.18% *versus* 58.97%; OR, 1.42 [95% CI, 1.12–1.81]; *p* = 0.004) and similar clinical safety compared to single-agent edaravone injection (Xu et al., 2021). Although edaravone dexborneol treatment showed superior clinical benefit, its cost-effectiveness in treating AIS awaits further investigation. To this end, this article evaluates the cost-effectiveness of edaravone dexborneol compared with edaravone for treating AIS patients from the Chinese healthcare payers’ perspective, aiming to inform policy and clinical practice.

## Materials and methods

### Model structure

**FIGURE 1:**
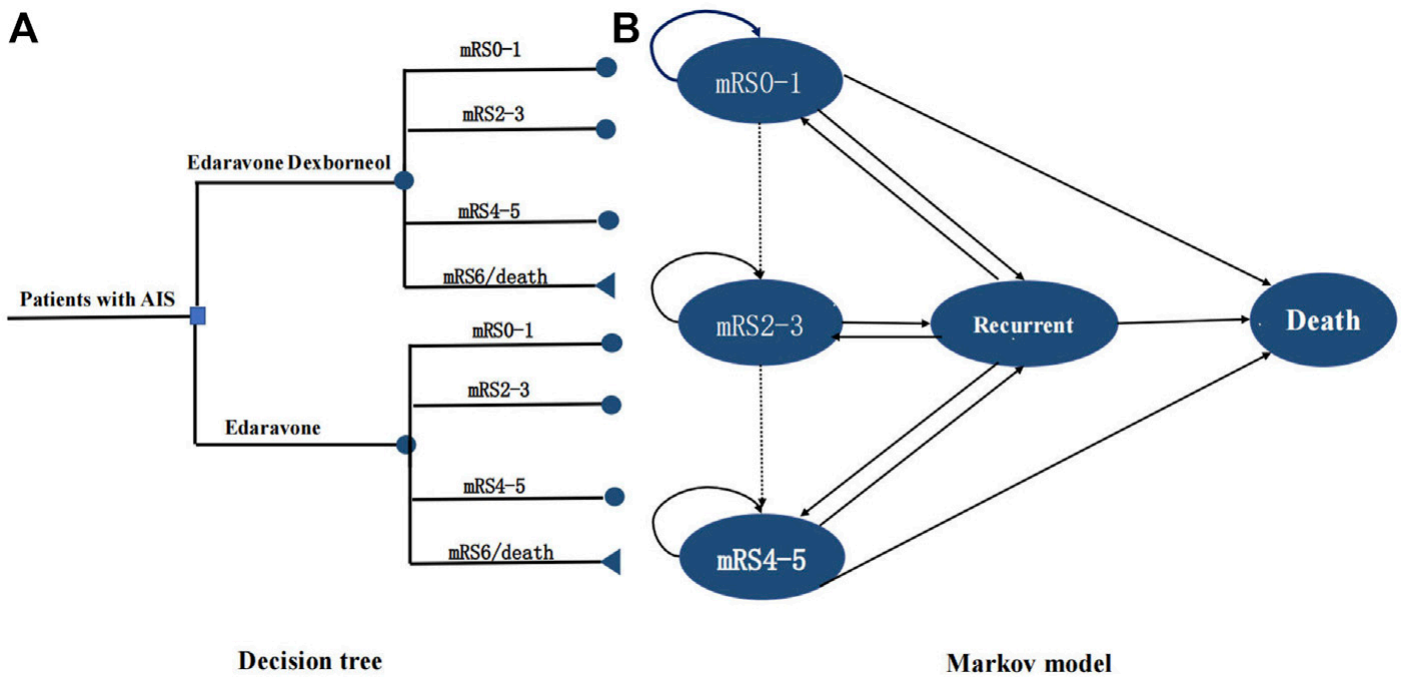
Model structure. **(A)** Short-term decision tree model and **(B)**. long-term Markov state transition model. A patient with an acute ischemic stroke within 48 h after symptom onset entered the model at 63 y old receiving either edaravone dexborneol or edaravone, and transitioned between health states until death or 30 y. Patients may have a stable health state, transition to a state of equal or greater disability after recurrent stroke, or died. mRS indicates modified Rankin Scale.

This study was conducted according to the latest Consolidated Health Economic Evaluation Reporting Standards (CHEERS 2022) reporting guidelines (Husereau et al., 2022). The decision tree and Markov model (Figure 1) were developed to simulate the 30-year cost-effectiveness of edaravone dexborneol *versus* edaravone. Our study was based on clinical data from the TASTE trial (NCT02430350), a phase III, randomized, double-blind, parallel, comparative study that enrolled 1,200 patients from May 2015 through December 2016 at 48 centers in China. With the development of disease, patients were assumed to transition among the four health states: no disability (mRS score 0–1), minor or moderate disability (mRS score 2–3), severe disability (mRS score 4–5), and dead (mRS score 6). Health states according to mRS categories at 3 months were derived from the clinical efficacy of the TASTE trial. Then, patients could undergo transitions annually for the remainder of their lifetime (30 years), and the cycle length was 1 year. At the end of each Markov cycle, patients either remained in their current health state, transitioned to a state of equal or greater disability due to a recurrent stroke, or died. A half-cycle correction was used for all states in every year.

We use incremental cost-effectiveness ratios (ICERs) to indicate cost-effectiveness and apply a willingness-to-pay (WTP) threshold of 1.5 times per capita GDP per QALY (121,464 CNY/QALY) (Cai et al., 2021). Both cost and effectiveness were discounted at a rate of 5% per year, as recommended by the China guidelines for pharmacoeconomic evaluations (China Market Press., 2020). We also report the net monetary benefits (NMBs), defined as expected QALY × WTP − cost, and this was used to compare strategies in the sensitivity analyses.

The model was developed using Microsoft Excel 2019.

### Patients and treatment

The target population in the model was patients diagnosed as AIS (aged 35–80 years), with a National Institutes of Health Stroke Scale (NIHSS) score of between 4 and 24, and administered within 48 h of AIS onset. The characteristics of the target population in this model were assumed to be the same as those in the TASTE study.

The intervention group received an edaravone dexborneol intravenous infusion of 37.5 mg/dose (edaravone, 30 mg; (+)-borneol, 7.5 mg), once every 12 h, which was continued for 14 days. The control group received an edaravone intravenous infusion of 30 mg/dose, once every 12 h, which was continued for 14 days.

### Clinical data and transition probabilities

The clinical efficacy parameters of the edaravone dexborneol and edaravone groups were derived from the TASTE study (Xu et al., 2021). In each cycle of the Markov model, patients could either remain in the same health state, experience a recurrent stroke, or die. Recurrent rates of stroke and mortality rates of recurrent strokes in years after the first 90 days were estimated by the China National Stroke Registry (CNSR) (Pan et al., 2014). Based on the previous study, we further assumed an increase in stroke recurrence rates by 1.019-fold per life year (Pan et al., 2014). Recurrent stroke stages were considered tunnel health states unless the onset led to death (Briggs et al., 2006). The age-specific death rate was drawn from and adjusted according to the causes of death in 2020 reported in the China Health Statistics Yearbook 2021 (National Bureau of Statistics of China, 2022a; National Health Commission of the People’s Republic of China, 2021). Excess mortality risk due to stroke was incorporated into the model as the hazard rate ratio for each mRS health state by using the age-specific death rate multiplied by the hazard rate ratio for each mRS health state (Samsa et al., 1999) (Table 1).

**TABLE 1:** Model input parameters.

Model input	Base case	Range	Distribution	Reference
Distribution of the modified Rankin scale (mRS) on day 90				TASTE study
Edaravone dexborneol				
mRS 0–1	67.2%	**—**	Dirichlet	Xu et al. (2021)
mRS 2–3	23.7%	**—**	Dirichlet	Xu et al. (2021)
mRS 4–5	7.8%	**—**	Dirichlet	Xu et al. (2021)
mRS 6	1.4%	**—**	Dirichlet	Xu et al. (2021)
Edaravone				
mRS 0–1	59.0%	**—**	Dirichlet	Xu et al. (2021)
mRS 2–3	28.1%	**—**	Dirichlet	Xu et al. (2021)
mRS 4–5	11.2%	**—**	Dirichlet	Xu et al. (2021)
mRS 6	1.7%	**—**	Dirichlet	Xu et al. (2021)
Odds ratio of mRS 0–1	1.42	1.12–1.81	—	Xu et al. (2021)
Transition probabilities				
Recurrent rate (per patient year)	0.12	0.1123–0.1241	Beta	CNS (Pan et al., 2014)
RR of recurrence per life year	1.019	1.014–1.024	Lognormal	CNSR (Pan et al., 2014)
Death rate with recurrent stroke	0.21	0.1887–0.2316	Beta	CNSR (Pan et al., 2014)
Non-stroke death hazard ratios				
mRS 0–1	1.00	1–1.2	Lognormal	Samsa et al. (1999)
mRS 2–3	1.19	1.1–1.3	Lognormal	Samsa et al. (1999)
mRS 4–5	2.04	1.4–3	Lognormal	Samsa et al. (1999)
Costs				
Edaravone dexborneol	4,099.20	3,279.36–4,099.2	Gamma	MENET
Edaravone	1,103.48	546–5,420.80	Gamma	MENET
Cost of intravenous injection	7.25	5-12	Gamma	12 provinces and cities
One-time hospitalization costs				
mRS 0–1	12,593.51	7,273.72–15856.24	Gamma	CNSR (Pan et al., 2014)
mRS 2–5	16,650.77	9,150.98–21,834.39	Gamma	CNSR (Pan et al., 2014)
mRS 6	14,269.82	6,705.26–18,861.18	Gamma	CNSR (Pan et al., 2014)
Post-hospitalization annual costs				
mRS 0–1	8,954.0	2,681.05–11420.88	Gamma	CNSR (Pan et al., 2014)
mRS 2–5	13,623.4	3,426.66–17131.99	Gamma	CNSR (Pan et al., 2014)
Recurrent cost	28,987.05	26,088.35–31,885.76	Gamma	Xiang et al. (2021)
Utilities				
mRS 0–1	0.80	0.80–0.95	Beta	Pan et al. (2014)
mRS 2–3	0.58	0.56–0.78	Beta	Pan et al. (2014)
mRS 4–5	0.28	0.05–0.36	Beta	Pan et al. (2014)
Recurrent stroke	0.34	0.27–0.42	Beta	Xiang et al. (2021)
Discount rate	5%	0%–8%	**—**	

### Cost

This study adopted the Chinese healthcare system’s perspective; therefore, only direct medical costs were considered, including the drug cost, cost of intravenous injection, hospitalization and post-hospitalization costs of AIS, and cost of recurrent stroke. Healthcare costs by mRS score were extracted from the China National Stroke Registry (CNSR) (Pan et al., 2014). The costs of edaravone dexborneol and edaravone were obtained from the mean bidding price from the Chinese drug bidding database on MENET (https://www.menet.com.cn/). The cost of intravenous injections was obtained from the mean price of medical services in 12 provinces across China to represent the national medical service price level (i.e., Beijing, Shanghai, Jiangsu, and Zhejiang). The costs of recurrent stroke were derived from the published literature (Xiang et al., 2021). All costs were discounted at 5% annually and inflated in 2021 Chinese Yuan 2020 using the medical care component of the consumer price index (Table 1).

### Utility

Utility values and weights were derived from the study and ranged from 0.36 for patients with an mRS of 4–5 to 0.84 for those with an mRS of 0–1 (Table 1) (Pan et al., 2014). Cumulative outcomes of treatment algorithms were measured by quality-adjusted life years (QALYs), which were discounted at an annual rate of 5%.

### Sensitivity analyses

Both deterministic sensitivity analyses and probabilistic sensitivity analyses were performed to ensure the robustness of the result. A one-way sensitivity analysis was performed to identify variables that significantly influence the INMB, including all variables within plausible ranges derived from the literature (Table 1). The uncertainty of effectiveness for edaravone dexborneol was calculated based on the proportions in the edaravone group and confidence intervals of the odds ratio of mRS 0–1 measured, as determined by the following formula: p2=(OR*p1)/(1+(OR−1)*p1). A tornado diagram was used to present the INMB by the one-way sensitivity analysis.

Furthermore, a Monte Carlo simulation was used to perform the probabilistic sensitivity analysis (PSA) with parameter inputs (efficacy, transition probabilities, costs, and utilities) sampled from fixed distributions. As transitions are multinomial, we assumed four mRS states followed the Dirichlet distribution, which is the multivariate generalization of the beta distribution with parameters equal to the number of categories in the multinomial distribution (Briggs et al., 2006). In addition, we assumed the probabilities and utilities followed a beta distribution, and costs followed a gamma distribution. The PSA performed 5,000 patient-level iterations, giving an incremental ICER scatterplot and a cost-effectiveness acceptability curve representing uncertainty.

### Ethics and engagement declaration

This research is entirely based on secondary data sources and does not contain any ethic-related issues. There was no engagement of patients, the general public, or stakeholders in the design of the study.

## Results

### Base case analysis

Table 2 shows the results in both the short term (1 and 5 years) and long term (30 years). In the base case analysis, the edaravone dexborneol treatment gained 0.03 (0.01–0.06) QALYs at an additional cost of CNY 2648.20 (1,429.64–3,493.64) in the first year, yielding an ICER of CNY 79703.94 per QALY gained. The treatment was cost-effective in the first year, using the threshold of CNY 121,464 (1.5× GDP per capita of China in 2021) as the WTP per QALY. However, the edaravone dexborneol treatment became highly cost-effective as the study time horizons extended. In the short term (5 years), the edaravone dexborneol treatment gained 0.11 (0.03–0.22) QALYs at an additional cost of CNY 1967.44 (-1,544.82–4,499.32), yielding an ICER of CNY 16905.45 per QALY gained. In the long term (30 years), the edaravone dexborneol treatment gained 0.25 (0.07–0.45) QALYs at an additional cost of CNY 2201.07 (-3,445.24–6,637.23), yielding an ICER of CNY 8823.41 per QALY gained. The NMB of edaravone dexborneol was higher than that of edaravone at a willingness-to-pay threshold of 121,464 CNY/QALY, indicating that this option was preferred on cost-effectiveness grounds.

**TABLE 2:** Cost-effectiveness results of edaravone *versus* edaravone dexborneol.

Time horizon		Edaravone	Edaravone dexborneol	Difference
1 year	Cost	25,395.46 (20,785.19–30767.30)	27,942.16 (23,421.34–33167.60)	2,648.20 (1,429.64–3,493.64)
	QALY	0.76 (0.67–0.83)	0.79 (0.70–0.87)	0.03 (0.01–0.06)
	ICER			79,703.94
	NMB	66,410.92 (55,181.63–77240.69)	67,899.91 (56,491.36–78679.01)	4,034.91 (-1835.78–5,409.41)
5 years	Cost	66,314.26 (52,030.52–84344.23)	68,180.20 (54,181.55–85198.40)	1967.44 (-1,544.82–4,499.32)
	QALY	2.60 (2.31–2.87)	2.71 (2.41–2.99)	0.11 (0.03–0.22)
	ICER			16,905.45
	NMB	248,970.44 (211,178.18–286249.97)	261,240.38 (222,177.83–297972.95)	12,269.94 (1,349.60–25366.18)
30 years	Cost	138,283.85 (103,852.12–181013.85)	140,383.92 (107,566.54–181688.71)	2,201.07 (-3,445.24–6,637.23)
	QALY	5.17 (4.56–5.80)	5.42 (4.79–6.04)	0.25 (0.07–0.45)
	ICER			8,823.41
	NMB	489,825.02 (405,543.00–574076.65)	518,025.14 (435,083.99–599258.74)	28,200.12 (6,797.43–53826.61)

### One-way sensitivity analysis

**FIGURE 2:**
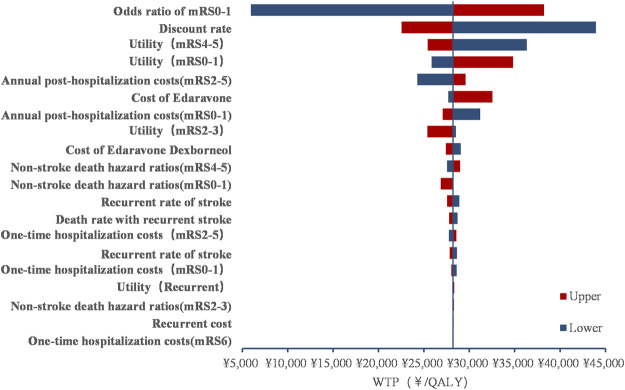
Tornado diagram.

As indicated in Figure 2, the most significant driver of the INMB was the odds ratio of mRS 0–1 at day 90, followed by the discount rate, the utility of mRS 4–5, and the utility of mRS 0–1. The sensitivity results for all assumptive inputs were under the 121,464 CNY/QALY thresholds, which demonstrated the robustness and consistency of the model outcomes.

### Probabilistic sensitivity analysis

**FIGURE 3:**
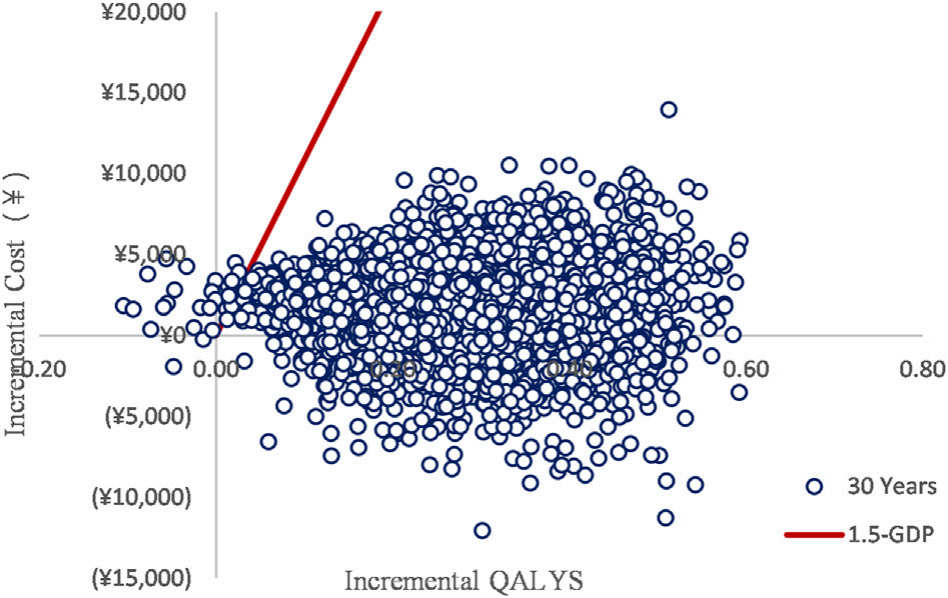
ICER scatterplot.

**FIGURE 4:**
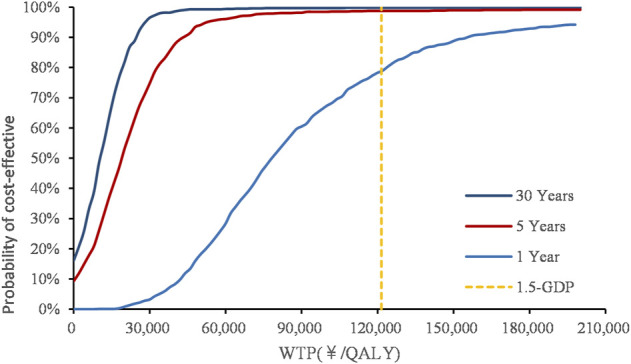
Cost-effectiveness acceptability curve.

The PSA with a 30-year time horizon is shown in Figure 3. With 5,000 iterations, edaravone dexborneol treatment was 99.5% cost-effective at a willingness-to-pay threshold of 121,464 CNY/QALY. Within the 5-year short run, the probability of edaravone dexborneol being the cost-effective strategy ranges from 76.3% (first year) to 98.90% (fifth year), which still holds a distinct advantage over edaravone. The cost-effectiveness acceptability curves of both treatments are shown in Figure 4.

## Discussion

As the evidence of reperfusion therapies keeps accumulating in recent years, the exploration of neuroprotective agents faces clinical translation dilemmas. In the last 10 years, a series of clinical trials of several neuroprotective agents, such as SAINT I and II trials (Lees et al., 2006; Shuaib et al., 2007; Diener et al., 2008), ALIAS trials (Ginsberg et al., 2013; Martin et al., 2016), URICO-ICTUS trials (Yu et al., 1998; Squadrito et al., 2000; Chamorro et al., 2014; Onetti et al., 2015), FAST-MAG trial (Saver et al., 2015), ACTION trial (Elkins et al., 2017), and ESCAPE-NA1 trial (Hill et al., 2020), have failed to show clinical efficacy. Of note, all the drugs have a specific target or show a well-proven inhibitory effect on the pathway in preclinical studies. However, for AIS, many pathways of damage in the ischemic cascade progress simultaneously and might interact with each other. Hence, combination treatments targeting several pathways of ischemic injury may have advantages over the single-pathway strategy.

Edaravone has been shown to relieve both endothelial and neuronal cell injury in AIS (Lapchak, 2010). (+)-Borneol has been shown to have potent neuroprotective effects through multiple molecular pathways in ischemia and reperfusion injury (Liu et al., 2011). Edaravone dexborneol—a novel neuroprotectant with an optimal proportion of 4:1—has been shown to have clear efficacy benefits compared with edaravone (Xu et al., 2021). Based on the TASTE study, this economic evaluation developed a model combining a decision tree and a Markov model to evaluate the cost-effectiveness of edaravone dexborneol *versus* edaravone in China from the perspective of the Chinese healthcare system. The main findings of this study suggest that compared with edaravone, the edaravone dexborneol strategy was highly likely to be a cost-effective treatment for Chinese patients treated with edaravone dexborneol within 48 h of AIS onset.

According to the present sensitivity analysis, the top factor with great influence on INMB was the odds ratio of mRS 0–1 at day 90, which is similar to the findings of Pan et al. (2014). Patients in mRS 0–1 status require less hospitalization and prognostic costs, so a treatment strategy that can improve the proportion of patients with mRS 0–1 after acute treatment will be more cost-effective. We also found that the economic advantage of the edaravone dexborneol strategy is increasing over time. The reason might be that the edaravone dexborneol strategy is highly effective in the functional outcome (mRS 0–1) for AIS, which dramatically influences the long-term utility and costs.

The China Stroke Prevention Programme Committee (CSPPC) was established in 2011 to improve the quality of stroke prevention and treatment. The CSPPC launched the accreditation process for stroke centers in 2015 and started publishing stroke emergency maps in 2016 (Chao et al., 2021; Ye et al., 2022). Although the rates of IVT and EVT in China are restricted by poor infrastructure, inefficient systems, and a deficiency of specialists, the significant differences in technical level and medical equipment across different grades of hospitals and the time points of patient entry into the hospital may cause inequity for those who cannot receive these technologies. Unlike IVT and EVT, using edaravone dexborneol was less restricted by other conditions of the health system and the time points of patient entry to the hospital. Therefore, the edaravone dexborneol was a cost-effective strategy to improve the outcomes of AIS patients. These findings inform national health care plans and implementation of AIS management in China.

The present study may have some limitations. First, our model focused on the influence of edaravone dexborneol for AIS, and functional status and costs as a result of other causes, such as congestive heart failure and myocardial infarction, were not included in the present model. Second, the utility data in this study were derived from literature studies, which are from the non-Chinese population. Third, this study was undertaken from the perspective of the Chinese healthcare system and did not include societal costs. However, given that the majority of the stroke population in the model has advanced age, the direct healthcare costs contribute far more than indirect costs. Fourth, adverse event costs were not calculated in this study due to the lack of corresponding adverse event cost data. However, given the low incidence of adverse events for both drugs, the impact of this cost on the final results is negligible Finally, patient adherence to secondary preventive treatment might have varied or decreased in the years after discharge, leading to underestimation or overestimation of the true cost-effectiveness of the intervention.

## Conclusion

This study demonstrated that edaravone dexborneol was highly likely to be a cost-effective alternative to treat AIS compared with edaravone in both short- and long-term economic analyses in China. These results provide important practical information for clinical practice decision-making and the resource allocation and policy development for reimbursement by medical insurance in China in the future.

## Data Availability

The original contributions presented in the study are included in the article/Supplementary Material; further inquiries can be directed to the corresponding author.
